# Etiology of Bright’s Disease

**Published:** 1873-06

**Authors:** F. C. Curtis

**Affiliations:** Albany, N. Y.


BUFFALO

### Medical and Surgical Journal.

VOL XII. JUNE, 1873. No. 11

### Original Communications.

:o:

ART. I.—*The Etiology of Bright's Disease.* By F. C. Curtis, M. D., of Albany. Read before the Albany County Medical Society, May 14th, 1873.

It has been a subject of remark at post mortem examinations that we very seldom see a perfectly normal kidney. Although the same might be said of most of the organs of the body, still it is true that Bright's disease is comparatively common.

The following case is peculiar only in regard to its etiology: Mr. L———, the patient, was between 45 and 50 years of age, a native of Canada, and by occupation a cooper. He had not, however, pursued his trade actively for some years, and had an easy life. He had always been healthy, and his family history is good. He was of light complexion and sanguine, nervous, temperament. There is no evidence that has ever been of scrofulous habit; neither has he been intemperate in the use of stimulants. He and his wife assert that he has always been perfectly well, excepting that he had attacks of sick headache, which were, as described by them, in no way peculiar. Aside from these he had no nausea or headache, his eyesight was normal, and he had no œdema. In fact the closest cross-questioning failed to detect any symptoms of disease of the kidneys prior to last January. The reason for questioning with particular care into the previous history of the patient was that he had an accident insurance policy, and his illness was asserted to be due to a fall.

Early in January, while walking in the street, he slipped on the ice and fell, striking on his back and the back of his head. His head was not particularly hurt, the fall seeming to cause only a general jarring of the body. He states that he was unconscious for a little time, after which he had severe pain in the head, which persisted. He also vomited several times in the course of the morning. The fall did not, however, disable him so but that he was able to procede directly to his business then in hand, which was of a pressing nature, being legal. He rode down town in an omnibus, walked to the lawyer's office, a short distance, and lay down for an hour or so, after which he attended to the business, and then rode home as he came. His headache continued and he vomited a few times, but was able to attend a trustee's meeting of his church, in the evening from 7½ till 9 o'clock, though feeling badly. His headache persisted for three or four days, and his face was bloated. His urine was noticed to be high colored on the third or fourth day. The urine was examined a week after the fall and found highly albuminous. Occasional vomiting continued for a week or two, coming in when the head ached especially.

The swelling of the face, about the eyes particularly, continued and about two weeks after the fall the limbs began to be œdematous. I saw him about the 1st of March for the first time. He then had no œdema of the face, nor did his face present the peculiar pallor of Bright's disease. He had some dyspnœa, but no cause for this was ascertained, as his heart and lungs were healthy, and there was no peritoneal or pleural effusion. His general condition was fair, and he was able to be about the house. There was no disturbance of vision, nor other nervous symptoms. His legs were œdematous. What his treatment was it is impossible to say, as he was under homoeopathic treatment. I examined his urine at this time, six or seven weeks after his fall, with the following result: Color a cherry red; transparent; no precipitate; sp. gr. 1022; albumen I should say about 5-6, as the test tube was solid with it. Microscopically there was found: renal epithelium; blood globules abundant; blood; epithelial and granular casts. It seemed pretty clear from the examination that the disease was acute and of short duration; rather than that it had been running a latent course, now for the first time developing.

The prognosis given at this time was that there was a possibility of recovery. The proportion of recoveries given by Frerichs from acute Bright's disease, is two-thirds of those attacked, and Roberts thinks this below the average, if cases resulting from scarlatina are included.

But the second examination of the urine, made three weeks later, seemed to settle the question of prognosis. The result of this was as follows: Urine a reddish color; opaque; reaction acid; sp. gr. 1025; albumen $\frac{3}{4}$:—microscopical appearance: precipitate not abundant; blood globules; renal epithelium, some of which was fatty; casts, which were abundant, were blood, epithelial, hyaline and fatty.

Evidently from this, the kidneys were passing beyond simple congestion and catarrh of the renal tubes. Here, within ten weeks of the outset, we see that there is fatty degeneration of the kidneys, and waxy casts indicating chronic disease. According to the view of Frerichs, Niemeyer, and the German pathologists generally—that the different anatomical changes of the kidneys are only stages of one disease,—the kidneys in this case had passed from the first to the second stage. Whether or not this unity of the forms of Bright's disease be a correct pathology, this case seems to illustrate very well its truth, to a certain extent at least. That the contracted, granular kidney is a third stage, ultimate to the large white kidney, is more difficult to prove. This patient did not live to show it; and in fact, I think from cases I have seen, patients are more apt to die having the large white kidney. I remember very well two cases of Bright's disease occurring side by side in the New York Hospital; one believed to have the "large white kidney," his symptoms being great œdema of the limbs, which was not relieved, although the urine was passed freely under diuretics; the pallor was very marked; symptoms of nervous disturbances presented, and he finally died of convulsions and coma. Here all the prominent symptoms of the affection were very marked. The other was considered a case of "small contracted kidney." He had no pallor, and no symptoms of nervous disturbance from first to last; nor was there any œdema, the only effusion being into the peritoneum, and subsequently into the pleural cavities. Under the use of muriated tincture iron and an occasional purge, he finally began to improve, the kidneys and skin acted freely, so the serous effusion disappeared entirely, albumen and casts cleared up from the urine, and to all appearance the man was cured and was so discharged from the Hospital.

In these two cases, presenting symptoms ascribed to the two pathological conditions, we see the one dying in the second stage (so called) while the other as far as could be seen, recovered. Many similar cases can be recalled by members of the Society. The second case related, moreover, never passed through the symptoms of the earlier stage, as seen in the first case. The theory of the unity of Bright's disease has been perhaps, gaining ground with us because of the many translations of valuable German works recently made; but I think the division into acute, smooth white, small granular, etc., made separate diseases, by Roberts, Dickinson, Wilks of Guy's Hospital, and others, has been the most commonly received here and in England.

The case in hand, from which I have made a digression, illustrates a partial reconciliation, showing a passage from a first stage to a second. The possibility of this, howerer, is not doubted that I know of, the difference being as to the second stage passing to the third, or, to use the terms of Stewart, from "fatty transformation" to "atrophy."

After the second examination of the urine, an unfavorable prognosis was given. The patient, however, at this time presented, clinically, an improved appearance. Later the œdema increased, the limbs, scrotum and penis swelled, he failed steadily and died without the occurrence of any symptoms of nervous disturbance, eighty-six days after the fall to which his death is attributed.

A post mortem examination was made next day. The parts were found œdematous, as above mentioned. There was no effusion into the serous cavities. The lungs were healthy. The heart was somewhat enlarged but the valves were normal and competent Abdominal organs healthy, except the kidneys. These were much enlarged, white, smooth and fatty. The capsule stripped up readily, leaving a smooth surface under it. The pyramids were partly destroyed. There were ecchymotic spots of small size in the left kidney. Microscopical examination showed abundance of fat, in the tubes, and fatty degeneration of the epithelium.

The termination of this disease fatally, within three months of the occurrence of its alleged cause, makes the etiology here especially important. Clearly the man died of Bright's disease simply. Had this been running a latent course and then developed at the time of injury—brought out by it perhaps? This could hardly be, for even though the urine had been free from albumen before this, other symptoms of ill-health must have been apparent, even though a diagnosis had been impossible. The urinalysis too, shows pretty conclusively that the inflammation of the kidneys was a recent one. Both subjective and objective symptoms point to about the first of January, as the probable time of the commencement of the disease.

The usual causes for Bright's disease, are exposure to cold, the use of alcoholics, and the influence of zymotic poisons—more especially scarlatina. Minor causes are hereditary tendency or constitution of body—such as scrofula and tubercle, and other diseases, excessive use of drugs, such as turpentine, copaiba, etc., pregnancy and other conditions.

I have searched through various authorities in regard to the etiology of this disease, and find them unanimous in giving these causes. Aitken alone alludes to certain causes which, to quote his words in full, "may be regarded as mechanical causes of irritation, but which so secondarily affect the constitution of a person predisposed to the disease, that Bright's disease rather than any other is the result—e. g., the irritation of *blows*, of cantharides or other irritants; the presence of calculi in the kidney," etc. The sentence is not a very clear one, but the idea seems to be that blows or other irritants may bring out the disease in a person predisposed to it by climate, constitution or habit of life.

We cannot find in this case, however, the existence of any predisposing cause. The accident occurred in steady winter weather, when it was not specially damp or variable; his occupation did not expose him to cold or moisture; his health was at its usual standard, and he had always been free from disease; his habits of life were in all respects temperate; the post mortem showed his other organs to be healthy.

The question of causation seems then to be limited to either the fall, acting simply and alone to produce the disease, or else the case is one of those in which the most searching analysis fails to detect the cause. The possibility of the fall acting as a cause for Bright's disease, unless effecting traumatic injury to the organ may well be doubted. There is a possibility in the case in hand, that injury was done to the kidneys not detected after death. But it is not easy to express an opinion in a matter so vague. Could reflex action, or injury to nerve centres act as a cause? It does not appear an impossibility, but I do not know any direct authority for it. I should be very glad to hear the views and experience of others in the matter.

